# The Role of Sodium-Glucose Cotransporter-2 Inhibitors in the Treatment of Polycystic Ovary Syndrome: A Review

**DOI:** 10.3390/jcm13041056

**Published:** 2024-02-13

**Authors:** Rachel Porth, Karina Oelerich, Mala S. Sivanandy

**Affiliations:** 1 Department of Medicine, Beth Israel Deaconess Medical Center, Boston, MA 02215, USA; rporth@bidmc.harvard.edu (R.P.); koeleric@bidmc.harvard.edu (K.O.); 2 PCOS Center, Division of Endocrinology, Beth Israel Deaconess Medical Center, Harvard Medical School, Boston, MA 02115, USA

**Keywords:** SGLT-2 inhibitors, polycystic ovary syndrome, treatment

## Abstract

Polycystic ovary syndrome (PCOS) is the most common endocrine disorder in reproductive-age women impacting their reproductive, mental, and metabolic health. Insulin resistance is a major driver of the pathophysiology of PCOS. There are several challenges with the management of this complex disorder including insufficient treatment options. Over the past 88 years, multiple hormonal and non-hormonal medications have been tried to treat the various components of this syndrome and there is no FDA (Food and Drug Administration)-approved medication specifically for PCOS yet. Sodium-glucose cotransporter-2 (SGLT-2) inhibitors have a unique mechanism of inhibiting the coupled reabsorption of sodium and glucose in renal proximal convoluted tubules. This review aims to examine the efficacy and side-effect profile of SGLT-2 inhibitors in patients with PCOS. In a limited number of studies, SGLT-2 inhibitors appear to be effective in improving menstrual frequency, reducing body weight and total fat mass, lowering total testosterone and DHEAS levels, and improving some glycemic indices in women with PCOS. SGLT2 inhibitors are generally well tolerated. With future research, it is possible that SGLT-2 inhibitors could become a key therapeutic option for PCOS.

## 1. Introduction

### 1.1. Polycystic Ovary Syndrome

Polycystic ovary syndrome (PCOS) is the most common endocrinopathy in women in the reproductive age group, with a worldwide prevalence of 10–13% depending upon the diagnostic criteria used. This disorder is also a common cause of infertility. The pathogenesis of PCOS involves genetic [1], epigenetic [2], neuroendocrine [3], reproductive, and metabolic alterations [4] and is not completely understood. It is hypothesized that aberrant activity of the GnRH (Gonadotropic-releasing hormone) pulse generator results in preferential secretion of the Luteinizing hormone over the Follicle-stimulating hormone, leading to increased androgen production in the theca cells and diminished testosterone-to-estrogen conversion in the granulosa cells of ovaries [5]. Insulin receptor binding defects and a decrease in Glut4 receptors lead to hyperglycemia and hyperinsulinemia, which further stimulates ovarian androgen production [6]. About 38–88% of women with PCOS are either overweight or obese [7], which substantially exacerbates insulin resistance and contributes to multiple other metabolic abnormalities. Though there are multiple commonly recognized phenotypes of PCOS (Type A, B, C, and D), insulin resistance and hyperinsulinemia are thought to be core features [8]. Insulin resistance is also a major driver of the pathophysiology of PCOS, leading to inflammation, metabolic complications, and reproductive dysfunction.

Management of PCOS includes treatment of oligo/anovulation, hyperandrogenic symptoms, and metabolic risks including prediabetes, type 2 diabetes, dyslipidemia, obesity, NAFLD, and obstructive sleep apnea [9]. Since polycystic ovary syndrome is associated with a multitude of symptoms, women with this disorder are often treated with more than one medication, both approved and off-label [10]. There are multiple treatment options and include hormonal preparations [11], antiandrogens, topical agents, laser photo epilation, electrolysis, diet, exercise, behavioral strategies, and insulin sensitizers. PCOS women are often treated with more than one medication and there is ongoing curiosity in finding novel treatment options to provide relief from the various issues associated with the syndrome.

Insulin resistance is a core component of PCOS, hence insulin sensitizers such as Metformin have been commonly used to treat overweight and obese women with PCOS to improve anthropometric and metabolic outcomes. In a review of 24 randomized controlled trials, metformin was associated with a reduction in body weight, BMI, fasting blood glucose, total testosterone, androstenedione, 17-hydroxyprogesterone levels, and an increase in the likelihood of pregnancy rate in women with PCOS [12]. It is equally effective as an active lifestyle intervention. The common side effects of metformin are gastrointestinal, such as nausea or abdominal discomfort, as well as B12 deficiency [13] in patients with pernicious anemia or who have undergone bariatric surgery. Other studies have found that metformin can very rarely increase the risk of lactic acidosis, particularly in patients who are taking high doses and have risk factors such as renal insufficiency or hepatic disease [14].

New medications such as GLP1 agonists and SGLT2 inhibitors have gained importance in treating patients with type 2 diabetes and PCOS in recent years. Metformin and SGLT2 inhibitors have the benefit of being administered orally compared to GLP1 agonists which are injectable except for oral semaglutide [15]. The GLP1 agonist semaglutide has been shown to cause weight loss and decrease HOMA-IR, basal insulin, and fasting blood glucose in obese patients with PCOS who have previously been unresponsive to lifestyle modifications [16]. Similarly, liraglutide also improved BMI, weight, and waist circumference in obese women with PCOS [17]. In a recent systematic review and meta-analysis, GLP1 agonists, alone or combined with metformin, appear to be more beneficial than metformin alone in reducing BMI, waist circumference, and insulin resistance in overweight and obese women with PCOS [18,19]. The focus of this review is to examine if SGLT2 inhibitors would have a meaningful role in treating PCOS.

### 1.2. SGLT2 Inhibitors

Sodium/glucose cotransporters (SGLT) are integral membrane proteins that mediate glucose transport across the cell membranes along with related substances and are hence called cotransporters [20]. SGLT-1 proteins are present in the cells lining the small intestine, facilitating absorption of D glucose and D galactose across the luminal brush border membrane. SGLT-2 proteins are predominantly expressed in the epithelial cells lining the proximal convoluted tubules (PCTs) of the kidneys and play a significant role in reabsorbing 90% of the glucose in the glomerular filtrate by lowering the renal threshold for glucose and cotransport sodium (Na+) [20]. The sodium-potassium-ATPase pump in the basolateral membrane of the epithelial cell in the PCT maintains the transport of sodium and glucose (via GLUT-2) back into the blood [20].

In the United States, Canagliflozin, Dapagliflozin, Empagliflozin, Ertugliflozin, and Bexagliflozin are the five FDA-approved SGLT2 inhibitors for treating adults with type 2 diabetes. Licogliflozin (LIK066), is a dual SGLT1 and 2 inhibitor [21]. By blocking the SGLT2 proteins in the proximal convoluted tubules of the kidneys, these medications reduce glucose reabsorption and increase urinary glucose excretion, leading to a reduction in HbA1c [22].

Apart from the above benefits, in a large randomized controlled study that enrolled 7020 patients (EMPA-REG OUTCOME), empagliflozin showed a 38% relative risk reduction in cardiac-related deaths in patients with type 2 diabetes at high risk for cardiovascular events [23]. A meta-analysis published by McGuire et al., showed improved cardiovascular and renal outcomes in type 2 diabetes patients on SGLT2 inhibitors, making it a drug of choice in patients with heart failure [24] and renal failure [25]. The cardioprotective effects of SGLT2 inhibitors in patients with heart failure with low ejection fraction appear to be related to improved endothelial function and vasodilatation, reduced inflammation, enhanced diuresis, and improved myocardial metabolism and efficiency [26]. This group of medications is currently being studied for the treatment of other metabolic disorders including obesity and non-alcoholic fatty liver disease.

## 2. SGLT2 Inhibitors in PCOS

Because prior research has shown such tremendous benefits for patients with other medical conditions who are started on an SGLT2 inhibitor, there has been an interest in exploring whether this may be a novel treatment option for patients with PCOS [27,28,29,30]. In this review, we examine the effects of SGLT2 inhibitors on various outcomes in women with polycystic ovary syndrome.

There have been five randomized clinical trials, Cai et al., 2022 [31], Elkind-Hirsch et al., 2021 [32], Javed et al., 2019 [33], Tan et al., 2022 [34], and Zhang et al., 2022 [35] that used SGLT2 inhibitors in women with PCOS and examined clinical outcomes, published as of end of year 2023. In all of these studies, overweight or obese patients with PCOS were treated with an SGLT2 inhibitor and they had at least one comparison arm. A systematic review and meta-analysis of the effect of these medications on the metabolic parameters in four of these five studies observed significant benefits in body weight, HOMA-IR, and fasting glucose level [30]. Table 1 shows the key characteristics of the randomized controlled trials that utilized SGLT2 inhibitors in women with PCOS.

## 3. Effects of SGLT2-Inhibitors on Various Outcomes

### 3.1. Effect on Menstrual Irregularity

Menstrual irregularity is one of the hallmarks of PCOS, and Metformin helps to induce ovulation in women with PCOS [36]. Both studies that evaluated the effects of SGLT2 inhibitors on menstrual patterns showed improvement, and one of the two showed statistically significant results. Cai et al. evaluated the impact of both metformin and SGLT2 inhibitors on menstrual irregularity, and at week 12, the number of menstrual cycles per year was increased in both the canagliflozin 1.34 (0.66 to 2.02) and the metformin group 1.37 (0.63 to 2.11), suggesting that canagliflozin is non-inferior to metformin. However, the results were not significant when comparing the two groups [31]. In Zhang et al., improvement in menstrual cycle irregularity was noted in both the canagliflozin/metformin group (80.95%, 17/21) and the metformin group (80.00%; 16/20); however, there was no significant difference seen between the two groups (*p* = 0.6228) [35].

### 3.2. Biochemical Hyperandrogenism

#### 3.2.1. Total Testosterone

Biochemical hyperandrogenism, elevation of testosterone, DHEAS, and/or androstenedione levels, is one of the diagnostic criteria in PCOS. Nearly 75% of women with PCOS have elevated androgen levels [37]. Combined oral contraceptive pills help to treat biochemical hyperandrogenism by suppressing ovarian androgen production and increasing the production of SHBG (sex hormone-binding globulin), which in turn decreases the free androgen levels. When looking at the effect of SGLT2 inhibitors on hormonal parameters, either alone or in combination with metformin, four out of the five studies showed a decrease in total testosterone [31,32,33,35] with two of the studies reaching statistical significance [32,35]. In Cai et al., there was an overall reduction in total testosterone in the canagliflozin group and no significant difference (*p* = 0.411) was seen between canagliflozin −0.15 (−0.38 to 0.08) vs. metformin −0.00 (−0.25 to 0.24) groups [31]. Similarly, Elkind-Hirsch et al. showed a statistically significant decrease in total testosterone (TT) when compared to baseline (*p* < 0.001) in the dapagliflozin as well as the other treatment groups including exenatide, exenatide/dapagliflozin, dapagliflozin/metformin, and phentermine/topiramate [32]. In Zhang et al., the canagliflozin/metformin combination decreased total testosterone significantly compared to metformin alone [canagliflozin/metformin: −2.49 ± 1.55 vs. metformin: −2.20 ± 1.30; (*p* = 0.0233)], though both groups demonstrated significantly lower TT levels when compared to baseline (*p* < 0.0001 and *p* = 0.0343, respectively) [35]. In Javed et al., TT levels increased in the empagliflozin group (% baseline change 2.6 (37.0) %) and decreased in the metformin group (% baseline change −14 (33.6)%); although, neither result was statistically significant [33]. Tan et al. demonstrated a non-significant reduction in total testosterone in the licogliflozin group compared to the placebo (9% reduction, 90% CI: 0.77–1.07; *p* = 0.340) [34]. Table 2 reviews the hormonal parameters examined in the studies that utilized SGLT2 inhibitors in women with PCOS.

#### 3.2.2. Free Androgen Index

In Zhang et al., in the canagliflozin/metformin group, the free androgen index (FAI) at 12 weeks decreased significantly compared to baseline (*p* = 0.0457) but not in the metformin-only group [35]. Additionally, Elkind-Hirsch et al. demonstrated that FAI (*p* < 0.001) significantly decreased in the SGLT2 inhibitor group. In Javed et al., there were non-significant reductions in FAI in both the metformin and empagliflozin groups (−7.0 ± 31.4% baseline change in the empagliflozin group vs. −9.7 ± 34.0% baseline change in the metformin group) [33]. In Tan et al., there was a non-significant reduction in FAI in the licogliflozin group compared to the placebo (21% reduction, 90% CI: 0.58–1.08, *p* = 0.204) [34].

#### 3.2.3. Dehydroepiandrosterone Sulfate

Three out of the four studies that evaluated Dehydroepiandrosterone sulfate (DHEAS) showed a decrease in levels [31,32,34]. One result was statistically significant compared to baseline [34], while one was statistically significant compared to metformin [31]. In Cai et al., there was a reduction in DHEAS levels in the canagliflozin group, but an increase in DHEAS levels in the metformin group (LS mean difference of −68.96 vs. 36.52, respectively, *p* = 0.013) [31]. In Tan et al., the licogliflozin group had a statistically significant decrease in DHEAS levels (effect size of 24%, treatment ratio of licogliflozin to placebo of 0.76 90% CI 0.65–0.89, *p* = 0.008) [34]. In Elkind-Hirsch et al., DHEA-S levels decreased in the SGLT2 inhibitor group when compared to baseline patient parameters; however, the drug effect did not reach statistical significance [32]. Both the empagliflozin and metformin groups in Javed et al. showed an increase in levels of DHEAS compared to baseline (1.0 ± 20.1 and 8.1 ± 15.0%, respectively) but were not statistically significant [33].

#### 3.2.4. Androstenedione

Four studies noted a decrease in androstenedione levels [31,33,34,35]. In Tan et al. the licogliflozin group showed a reduction in androstenedione levels compared to the placebo (effect size of 19%, treatment ratio of licogliflozin to placebo of 0.81 90% CI 0.68–0.99, *p* = 0.089) [34]. In Javed et al., the empagliflozin group had an overall decrease in androstenedione levels (−2.2 (24.4)% baseline change) while the metformin group had an overall increase in levels (5.6 (59.8)% baseline change) [33]. In Cai et al., there was a decrease in androstenedione levels in the canagliflozin group, while levels slightly increased in the metformin group; although neither was statistically significant (−0.48 (−1.04 to 0.09) vs. 0.04 (−0.49 to 0.56) *p* = 0.199) [31]. In Zhang et al., no significant changes in androstenedione were seen in either the canagliflozin/metformin group, or in metformin alone compared to the baseline [35].

#### 3.2.5. Sex Hormone-Binding Globulin (SHBG)

Two out of five studies that examined SHBG showed a significant increase in levels [32,33].

In Zhang et al., in the metformin group the SHBG (sex hormone-binding globulin) levels increased significantly (*p* = 0.0303), but no changes were observed in the canagliflozin/metformin group [35]. In Javed et al., there was a significant increase in the SHBG levels in the empagliflozin group (9.9 ± 22.6% baseline change, *p* = 0.049) compared to the baseline [33]. The metformin group showed a non-significant increase in levels of SHBG (6.4 ± 25.5%) [33]. In Elkind-Hirsch et al., SHBG levels significantly increased in the dapagliflozin group (*p* < 0.001) [32]. In Cai et al., SHBG levels decreased in both the canagliflozin and metformin groups with no significant difference between the two groups (−4.82 (−19.40 to 9.75) vs. −13.58 (−31.21 to 4.05) *p* = 0.472) [31]. In Tan et al., there was a non-significant increase in SHBG levels in the licogliflozin group compared to the placebo (treatment ratio of licogliflozin to placebo of 1.15 90% CI 0.97–1.36, *p* = 0.173) [34].

### 3.3. Obesity and Other Anthropometric Indices

Nearly 38–88% of women with PCOS have a BMI in the overweight or obese range [38]. Metformin improves metabolic outcomes in PCOS women and is therefore recommended by international guidelines for women with a BMI > 25 [9]. SGLT2 inhibitors, just like metformin, appear to improve anthropometric indices, with multiple studies showing a decrease in BMI, waist circumference, and hip circumference. In Cai et al., all study participants had a similar decrease in weight (kg) [canagliflozin: −2.82 (−3.97 to −1.66) vs. metformin: −2.68 (−3.93 to −1.43)] and BMI (kg/m^2^) [canagliflozin: −1.04 (−1.56 to −0.53) vs. metformin: −0.90 (−1.46 to −0.35)] [31]. Similarly, Javed et al. did not find a statistically significant change in total mass (*p* = 0.079) or BMI (*p* = 0.069) [33]. In Elkind-Hirsch et al., patients in the exenatide/dapagliflozin and phentermine/topiramate groups had greater decreases in BMI compared to patients in other treatment groups [32]. In Zhang et al., there was a statistically significant decrease in both body weight and BMI for patients receiving both canagliflozin and metformin (*p* < 0.0001 and <0.0001, respectively) as well as metformin alone (*p* < 0.0001 and *p* < 0.0001, respectively), but not a statistically significant difference between groups [35]. Tan et al. found that body weight before and after treatment was stable in patients who received licogliflozin (BMI not examined) [34]. Table 3 shows the studies that examined the effects of SGLT2 inhibitors in women with PCOS.

Three studies examined waist and hip circumference. Cai et al. found similar decreases for canagliflozin and metformin in waist circumference (cm) [canagliflozin: −4.05 (−6.18 to −1.91) vs. metformin: −3.27 (−5.54 to −0.99) (*p* = 0.629)] and hip circumference (cm) [canagliflozin: −2.62 (−4.02 to −1.21) vs metformin: −2.93 (−4.42 to −1.44) (*p* = 0.767)] [31]. Elkind-Hirsch et al. only found a statistically significant decrease in waist circumference and waist-to-hip ratio with exenatide plus dapagliflozin and phentermine plus topiramate compared to dapagliflozin with metformin [32]. Finally, Javed et al. found that empagliflozin led to a statistically significant decrease in waist circumference (*p* = 0.024) and hip circumference (*p* = 0.013) compared to the baseline. There was also a statistically significant difference between empagliflozin and metformin for both waist (empagliflozin: −1.6 ± 2.8% vs. metformin: 0.2 ± 2.1%; *p* = 0.029) and hip circumference (empagliflozin: −2.0 ± 3.0% vs. metformin: 1.1 ± 1.9%; *p* = 0.001) [33].

### 3.4. Effect on Glycemic Indices

Nearly 20–35% of women with PCOS have impaired glucose tolerance and 5–10% have type 2 diabetes [39]. Three out of four studies that examined glycemic indices appear to show statistically significant findings and improvements in insulin resistance. Table 4 reviews measures of glycemic control across four studies. Cai et al. found that canagliflozin led to an increase in homeostatic model assessment insulin sensitivity index (HOMA-ISI) [LS mean 0.42 95% CI (0.23 to 0.62)], along with a decrease in glycated hemoglobin (HbA1c) [LS mean −0.26 95% CI (−0.43 to −0.09)], fasting blood glucose [LS mean −0.23 95% CI (−0.40 to −0.06)], fasting serum insulin [LS mean −7.70 95% CI (−11.46 to −3.94), and postprandial insulin [LS mean –81.43 95% CI (−122.71 to −40.14)] [31]. Tan et al. showed that licogliflozin reduced hyperinsulinemia by decreasing the area under the curve for insulin by 68%, the maximum peak of insulin by 74%, HOMA-IR by 30%, and fasting glucose by 6% [34]. Zhang et al. found a lower area under the curve for glucose and the area under the curve for the insulin-to-glucose ratio in patients who received metformin + canagliflozin vs. metformin alone. However, there were no differences in fasting blood glucose, fasting insulin, area under the curve for insulin, or HOMA-IR [35]. Javed et al. did not find that empagliflozin leads to any changes in insulin sensitivity (insulin, fasting glucose, HOMA-IR) [33].

### 3.5. Changes in Metabolic Indices

In a limited number of studies, SGLT2 inhibitors have also shown mixed results in impacting metabolic parameters, as seen in Table 5. One out of the four studies that looked at cholesterol found statistically significant decreases in total cholesterol and triglycerides from the baseline with canagliflozin and metformin [35], and one out of two studies that examined blood pressure found statistically significant improvements in systolic (SBP) and diastolic blood pressure (DBP) [32]. In Cai et al., canagliflozin was not found to significantly decrease total cholesterol (mmol/L) [0.17 (−0.05 to 0.39)], LDL cholesterol (mmol/L) [0.22 (0.06 to 0.51)], or increase HDL cholesterol [0.02 (−0.17 to 0.13)]. It did lead to a slight decrease in triglycerides (mmol/L) [−0.36 (−0.54 to −0.17) [31]. Zhang et al. and Javed et al. did not find significant differences in lipid parameters [33,35]. Elkind-Hirsch et al. found that triglycerides were reduced with exenatide/dapagliflozin, but not with dapagliflozin alone [32]. Data on the impact of SGLT2 inhibitors on systolic and diastolic blood pressure are also limited. Out of the five studies reviewed, only two (Elkind-Hirsch et al. [32] and Javed et al. [33]) looked at changes in SBP and DBP. While Elkind et al. found that systolic and diastolic blood pressures were both significantly decreased by all treatments (exenatide, dapagliflozin, exenatide/dapagliflozin, dapagliflozin/metformin, and phentermine/topiramate) (*p* < 0.035) [32], they did not assess differences between treatment groups. Javed et al. found no differences in blood pressure after 12 weeks in patients on empagliflozin compared to metformin [33]. It is important to note that patients in both studies were normotensive before starting the study.

### 3.6. Side Effects of SGLT2 Inhibitors and Limitations of the RCTs

While SGLT2 inhibitors can have many benefits for patients with PCOS, potential adverse effects must be noted. The side effect profile of SGLT2 inhibitors in these studies appears to be favorable, with primarily mild gastrointestinal symptoms like metformin. The most common adverse effects reported by the studies overall included yeast infections, urinary tract infections, lightheadedness, diarrhea, and flatulence [32,34]. In Cai et al., adverse effects were more common in the metformin group compared to the SGLT2 inhibitor group [31]. In Zhang et al., the most common side effects in the canagliflozin/metformin group were nausea, diarrhea, dizziness, and abdominal pain [35]. Though no serious adverse events were reported in any of the studies, patients with PCOS trialing an SGLT2 inhibitor should be aware of potential side effects, most commonly the gastrointestinal and potentially infectious.

In studies of patients with type 2 diabetes, the use of SGLT2 inhibitors has been associated with an elevated risk of diabetic ketoacidosis (DKA) [40]. In particular, patients are at increased risk of euglycemic DKA or moderately elevated glucose levels. There are two mechanisms behind this described by Liu et al. [41]. First, as SGLT2 inhibitors increase the excretion of urinary glucose, there is a decrease in insulin secretion which can lead to the production of free fatty acids and conversion to ketone bodies, and second, there is also an increase in the secretion of glucagon, leading to the increased production of ketone bodies [41].

Data from the CANVAS Program, which looked at patients with type 2 diabetes and high cardiovascular risk, revealed an increase in the rate of fractures in patients taking SGLT2 inhibitors. In this study, patients taking an SGLT2 inhibitor had a greater rate of all fractures compared to the placebo, as well as a similar trend with low-trauma fractures [42]. One caveat is that most of the research in the literature on SGLT inhibitors has been done on patients with diabetes, and more studies are needed in patients with PCOS to evaluate the side-effect profile in this population.

Because all the patients in the studies that used SGLT2 inhibitors were either overweight or obese, more research is needed to determine if SGLT2 inhibitors are effective for PCOS women with a normal BMI. Another potential limitation was the variability in study length, which was from 2 to 24 weeks. Though all the studies investigated various biochemical parameters, no data were provided regarding clinical hyperandrogenic factors (i.e., hirsutism, acne, alopecia), which would be helpful to assess in further studies. In addition, pregnancy rates and outcomes were not evaluated in these studies.

## 4. Conclusions

Polycystic ovary syndrome is the most common hormonal disorder in women of childbearing age. Though there is no cure for PCOS, the goal is to manage the symptoms and prevent long-term complications. Insulin resistance and compensatory hyperinsulinemia affect a significant percentage of women with PCOS. SGLT2 inhibitors, by improving glucotoxicity and insulin sensitivity, could play a beneficial role in treating some of the metabolic and hormonal derangements in PCOS. They have been shown to improve menstrual frequency, reduce body weight and total fat mass, lower total testosterone and DHEAS levels, and improve glycemic indices in patients with PCOS. SGLT2 inhibitors, when compared to a placebo or standard of care in patients with PCOS, appear to have a similar effect on improving menstrual cycles as metformin. Given the benefits shown in these studies, SGLT2 inhibitors could be considered a novel treatment strategy for PCOS as they target multiple hormonal, metabolic, and glycemic control-associated abnormalities. Alternatively, for patients who are unable to tolerate or have contraindications to metformin, SGLT2 inhibitors have shown promise to be a viable alternative. Given the limited number of studies that have investigated the benefits of SGLT2 inhibitors in patients with PCOS, larger sample sizes are needed to further evaluate these benefits. In particular, randomized double-blind placebo-controlled trials comparing metformin, SGLT2 inhibitors, and a placebo would be helpful in further distinguishing metabolic, hormonal, glycemic, and clinical outcomes.

## Figures and Tables

**Table 1:** Key characteristics of the randomized controlled trials that utilized SGLT2 inhibitors in women with PCOS.

	Cai et al. [31]	Elkind-Hirsch et al. [32]	Javed et al. [33]	Tan et al. [34]	Zhang et al. [35]
SGLT2 inhibitor	Canagliflozin 100 mg daily	Dapagliflozin 10 mg daily	Empagliflozin 25 mg daily	Licogliflozin 50 mg three times daily	Canagliflozin 100 mg daily (with Metformin 1000 mg twice a day)
Comparison Arm	M	E, E/D, D/M, P/T	M	P	M
Number of participants	C: 33 M: 35	E: 20 D/E: 20 D: 17 D/M: 19 PH/T:16	EM: 19 M: 20	L: 10 P: 10	C/M: 21 M: 20
Study duration (weeks)	12	24	12	2	12
BMI (kg/m^2^)	C: 27.26 (25.55 to 28.99) M: 27.95 (26.22 to 29.69)	E: 38.6 ± 1.1 E/D: 39.9 ± 0.9 D: 38 ± 1.1 D/M: 37.6 ± 1.1 P/T: 38.4 ± 1.1	EM: 37.1 ± 6.2 M: 38.7 ± 7.8	38.1 ± 6.3	C/M: 31.11 ± 3.02 M: 29.33 ± 3.19
Age (years)	C: 28.58 (26.72 to 30.43) M: 27.83 (25.97 to 29.68)	E: 30 ± 1.1 E/D: 31 ± 1.4 D: 28 ± 1.5 D/M: 31 ± 1.6 PH/T: 30 ± 1.5	EM: 26.0 (8.0) M: 31.5 (20.0)	27.6 ± 5.3	C/M: 26.38 ± 5.89 M: 25.5 ± 4.36

C = canagliflozin; M = metformin; D = dapagliflozin; EM = empagliflozin; L = licogliflozin; E = exenatide; PH = phentermine; T = topiramate; P = placebo.

**Table 2:** Comparison of the Changes in Hormonal Parameters in the trials that used SGLT2 inhibitors in women with PCOS.

	Cai et al. [31]	Elkind-Hirsch et al. [32]	Javed et al. [33]	Tan et al. [34]	Zhang et al. [35]
SGLT1/2 inhibitor	Canagliflozin	Dapagliflozin	Empagliflozin	Licogliflozin	Canagliflozin (with Metformin)
Total Testosterone	Pre-treatment: 1.78 ng/mL (1.52 to 2.05) LS mean −0.15 (−0.38 to 0.08)	Pre-treatment: 46 ng/dL ± 5 Post-treatment: 35 ng/dL ± 4.4 (−11) *	Pre-treatment: 1.6 nmol/L ± 0.4 Post-treatment: 1.6 nmol/L ± 0.6	Effect size: 9% decrease in (TR_LIK066_:TR_PCB_ [TT]: 0.91; 90% CI: 0.77–1.07; *p* = 0.340)	Pre-treatment: 0.95 ng/mL (0.78–1.08) Post-treatment: 0.53 ng/mL (0.45–0.84) *
Free Androgen Index	n/a	Pre-treatment: 6.7 ± 1.0 Post-treatment: 4.7 ± 0.8 (−2.0) *	Pre-treatment: 10.3 ± 3.0 Post-treatment: 9.4 ± 3.6	n/a	Pre-treatment: 28.62% ± 16.4 Post-treatment: 19.15% ± 13.19 *
DHEAS	Pre-treatment: 261.80 ug/dL (217.77 to 305.83) LS mean −68.96 (−126.36 to −11.55) **	Pre-treatment: 210 mcg/dL ± 22 Post-treatment: 187 mcg/dL ± 24 (−23)	Pre-treatment: 6.1 μmol/L ± 1.6 Post-treatment: 6.2 μmol/L ± 2.1	Effect size: 24% decrease in (TR_LIK066_:TR_PCB_ [DHEAS]: 0.76; 90% CI: 0.65–0.89; *p* = 0.008 *	n/a
Androstenedione	Pre-treatment: 4.17 ng/mL (3.53 to 4.81) LS mean −0.48 (−1.04 to 0.09)	n/a	Pre-treatment: 5.7 nmol/L ± 1.4 Post-treatment: 5.7 n μmol/L ± 1.9	Effect size: 19% decrease in (TR_LIK066_:TR_PCB_ [A4]: 0.81; 90% CI: 0.68–0.99; *p* = 0.089)	Pre-treatment: 3.57 ng/mL ± 1.29 Post-treatment: 3.22 ng/mL ± 1.35
SHBG	Pre-treatment 33.76 nmol/L (21.64 to 45.89) LS mean −4.82 (−19.40 to 9.75)	Significantly increased *—no data provided by paper	Pre-treatment: 17.3 nmol/L ± 6.4 Post-treatment: 19.2 nmol/L ± 8.5 *	Effect size: 15% increase in (TR_LIK066_:TR_PCB_ [SHBG]: 1.15; 90% CI: 0.97–1.36; *p* = 0.173)	Pre-treatment: 13.60 nmol/L (8.55–20.15) Post-treatment: 13.6 nmol/L (9.55–24.10)
Free testosterone	Pre-treatment 2.40 pg/mL (1.92 to 2.88) LS mean 0.30 (−0.30 to 0.89)	n/a	n/a	Effect size: 12% decrease in (TR_LIK066:_TR_PCB_[FT}: 0.88; 90% CI: 0.70–1.11; *p* = 0.353)	n/a

DHEAS—dehydroepiandrosterone sulfate; SHBG—sex hormone-binding globulin; FT—free testosterone; A4—androstendione; TR_LIK066_:TR_PCB_—ratio of relative changes between licogliflozin and placebo; * statistically significant within-group comparison *p* < 0.05; ** statistically significant compared to the metformin alone group in Cai et al. To convert from ng/dL to nmol/L multiply ng/dL by 0.0347. μIU/mL is equivalent to mU/L. To convert ng/mL to nmol/L multiply the ng/mL by 2.5. To convert μg/dL to μmol/L multiply the μmol/L by 20.7. μg/dL is equivalent to mcg/dL.

**Table 3:** Comparison of the changes in anthropometric parameters and BMI in studies that used SGLT2 inhibitors in women with PCOS.

	Cai et al. [31]	Elkind-Hirsch et al. [32]	Javed et al. [33]	Zhang et al. [35]
SGLT2 inhibitor	Canagliflozin	Dapagliflozin	Empagliflozin	Canagliflozin (with Metformin)
BMI (kg/m^2^)				
Pretreatment	27.26 (25.55 to 28.99)	38 ± 1.1	37.1 ± 6.2	31.11 ± 3.02
Posttreatment	LS Mean: −1.04 (−1.56 to −0.53)	37.4 ± 1.2 (−0.6) *	36.6 ± 6.0	28.62 ± 2.91 *
Weight (kg)				
Pretreatment	72.94 (67.89 to 77.99)	104 ± 3	102.3 ± 16.6	81.23 ± 9.83
Posttreatment	LS Mean: −2.82 (−3.97 to −1.66)	102.6 ± 4 (−1.4) *	101.5 ± 16.3	75.40 ± 8.68 *
Waist Circumference (cm)				
Pretreatment	92.87 (88.00 to 97.75)	104 ± 3	101.2 ± 9.7	n/a
Posttreatment	LS Mean: −4.05 (−6.18 to −1.91)	101 ± 3.2 (−3)	99.6 ± 9.5 *	
Waist-to-hip ratio				
Pretreatment	0.91 (0.88 to 0.93)	0.81 ± 0.02	n/a	n/a
Posttreatment	LS Mean: −0.02 (−0.04 to 0.00)	0.79 ± 0.017 (−0.02)	n/a	n/a

* *p* < 0.05.

**Table 4:** Changes in glycemic indices in the trials that employed SGLT2 Inhibitors in women with PCOS.

	Cai et al. [31]	Elkind-Hirsch et al. [32]	Javed et al. [33]	Zhang et al. [35]
SGLT2 inhibitor	Canagliflozin	Dapagliflozin	Empagliflozin	Canagliflozin (with Metformin)
Hemoglobin A1c (HbA1c)%				
Pretreatment	5.60 (5.37 to 5.83)	n/a	n/a	n/a
Posttreatment	Change from baseline LS mean: −0.26 (−0.43 to −0.09)	n/a	n/a	n/a
HOMA-IR				
Pretreatment	5.33 (3.92 to 6.73)	4.1 ± 0.7	2.6 (2.1)	5.70 (3.38–6.08)
Posttreatment	Change from baseline LS mean: −2.04 (−2.89 to −1.18)	3.4 ± 0.6 (−0.7) *	2.4 (2.7)	3.14 (1.91–4.71) *
FBG				
Pretreatment	5.18 mmol/L (4.99 to 5.38)	98 mg/dL ± 2.3	4.5 mmol/L (0.6)	5.70 mmol/L (5.27–6.02)
Posttreatment	Change from baseline LS mean: −0.23 (−0.40 to −0.06)	93 mg/dL ± 2.1 (−5) *	4.5 mmol/L (0.6)	5.20 mmol/L (4.88–5.35)
FINS				
Pretreatment	22.58 mU/L (16.99 to 28.17)	n/a	12.6 μIU/mL (11.6)	21.5 mU/L (14.35–24.20)
Posttreatment	Change from baseline LS mean: −7.70 (−11.46 to −3.94)	n/a	12.7 μIU/mL (14.4)	12.0 mU/L (8.20–20.15) *

HOMA-IR—homeostatic model assessment of insulin resistance; FBG—fasting blood glucose; FINS—fasting serum insulin. * *p* < 0.05; [31]. To convert from mg/dL to mmol/L divide mg/dL by 18. μIU/mL is equivalent to mU/L.

**Table 5:** Changes in metabolic parameters in the studies that used SGLT2 inhibitors in women with PCOS.

	Cai et al. [31]	Elkind-Hirsch et al. [32]	Javed et al. [33]	Zhang et al. [35]
SGLT2 inhibitor	Canagliflozin	Dapagliflozin	Empagliflozin	Canagliflozin (with Metformin)
Total Cholesterol				
Pretreatment	4.87 mmol/L (4.58 to 5.16)	183 mg/dL ± 6	4.8 mmol/L ± 1.0	4.90 mmol/L ± 0.93
Posttreatment	LS mean: 0.17 mmol/L (−0.05 to 0.39)	186 mg/dL ± 11 (+3.0)	4.7 mmol/L ± 1.1	4.54 mmol/L ± 0.80 *
Triglycerides				
Pretreatment	1.75 mmol/L (1.37 to 2.14)	143 mg/dL ± 21	1.5 mmol/L (1.3)	1.54 mmol/L (1.09–2.01)
Posttreatment	LS mean: −0.36 mmol/L (−0.54 to −0.17)	n/a	1.4 mmol/L (0.9)	1.20 mmol/L (0.84–1.63) *
LDL				
Pretreatment	3.04 mmol/L (2.66 to 3.43)	107 mg/dL ± 6	2.8 mmol/L ± 1.0	3.06 mmol/L ± 0.97
Posttreatment	LS mean: 0.22 mmol/L (0.06 to 0.51)	113.5 mg/dL ± 10 (6.5)	2.7 mmol/L ± 1.1	2.83 mmol/L ± 0.70
HDL				
Pretreatment	1.33 mmol/L (1.12 to 1.54)	44 mg/dL ± 2	1.1 mmol/L ± 0.2	-
Posttreatment	LS mean: 0.02 mmol/L (−0.17 to 0.13)	43 mg/dL ± 2.2 (−1.0)	1.1 mmol/L ± 0.2	-

* *p* < 0.05; [31]. To convert from mg/dL to mmol/L divide mg/dL by 18.
